# Post-Li batteries: promises and challenges

**DOI:** 10.1098/rsta.2018.0297

**Published:** 2019-07-08

**Authors:** Alexandre Ponrouch, M. Rosa Palacín

**Affiliations:** 1 Institut de Ciència de Materials de Barcelona (ICMAB-CSIC), Campus UAB, 08193 Bellaterra, Catalonia, Spain; 2 ALISTORE-ERI European Research Institute, France

**Keywords:** sodium batteries, calcium batteries, magnesium batteries, metal anodes

## Abstract

Current societal challenges in terms of energy storage have prompted an intensification in the research aiming at unravelling new high energy density battery technologies. These would have the potential of having disruptive effects in the world transition towards a less carbon-dependent energy economy through transport, both by electrification and renewable energy integration. Aside from controversial debates on lithium supply, the development of new sustainable battery chemistries based on abundant elements is appealing, especially for large-scale stationary applications. Interesting alternatives are to use sodium, magnesium or calcium instead of lithium. While for the Na-ion case, fast progresses are expected as a result of chemical similarities with lithium and the cumulated Li-ion battery know-how over the years, for Ca and Mg the situation is radically different. On the one hand, the possibility to use Ca or Mg metal anodes would bring a breakthrough in terms of energy density; on the other, development of suitable electrolytes and cathodes with efficient multivalent ion migration are bottlenecks to overcome.

This article is part of a discussion meeting issue ‘Energy materials for a low carbon future’.

## 1. Introduction

World transition towards a less (or non!) carbon-dependent energy economy and technology is urged for, and will need to change transport by electrification and integrate more renewable energy to the grid. This can only be achieved by widespread deployment of energy storage and conversion at large (grid) and intermediate (vehicle) scales. The worldwide rechargeable battery market is continuously growing and the cost of the state-of-the-art electrochemical energy storage technology, the Li-ion battery (LIB), has been reduced by 8% annually in the last decade at the pack level [1], and it is now reaching its fundamental limits in terms of energy density. Moreover, the risks of limited lithium supply and/or significantly increased prices cannot be ignored [2]. Consequently, innovative sustainable battery chemistries based on other elements and enabling higher energy densities must be developed, in line with recent ranking of next-generation batteries as the no. 2 game-changing technology by the World Economic Forum in Davos 2016. The quest for next-generation batteries must be based on a rational approach targeting long-term sustainable solutions by using abundant materials and eco-efficient production protocols. If this can be done reducing cost, it will also allow a wider market penetration and thereby account for socio-economic factors and employed to societies at a global scale.

The aim of increasing the energy density of batteries is a long-lasting game with rather simple rules. One needs to (i) increase the cell voltage (difference in working potentials between the positive and the negative electrode active materials), (ii) increase the specific capacity of the positive and negative electrode active materials, and/or (iii) decrease the dead weight of the cell (separator, electrolyte, cell packaging, etc.). While significant progress was made over the last decades in order to improve the cell engineering, the quest for new battery chemistries (anode and cathode active materials) is advancing at a slower pace.

**Figure 1.**
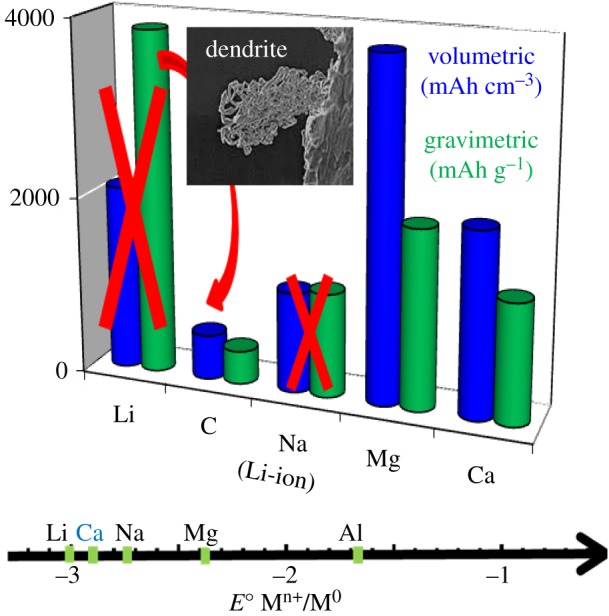
Gravimetric/volumetric capacities and standard reduction potential of metal anodes compared to conventional LIBs. The energy density is the product of both measures. Reprinted from [3] with permission of the Electrochemical Society. (Online version in colour.)

The ‘holy grail’ in the battery community is to build rechargeable cells using Li metal as an anode. Indeed, being the lightest metal with a low standard potential (−3.04 V versus normal hydrogen electrode), it results in very high specific capacity (figure 1). Intensive research on battery concepts based on lithium metal anodes such as Li–S and Li–air technologies have despite large theoretical promise, proven many intrinsic issues related to safety, e.g. dendritic growth of Li and mechanistic bottlenecks, e.g. polysulfide dissolution or reactivity of radicals to be showstoppers [4–6]. Sodium metal, as lithium, has also a tendency to dendritic growth [7]. This fact coupled to its relatively low melting point (approx. 98°C) makes it an even less safe anode. However, magnesium or calcium seem to be less prone to dendrite formation, potentially due to the lower self-diffusion barriers of the adatom during plating [8–10]. The use of these light metals couples the advantages of high theoretical volumetric capacity (figure 1) with natural abundance, low cost and safety. Furthermore, the use of divalent charge carriers accounts for a twofold increase in achievable energy density with respect to Li^+^ for equal amounts of reacted ions.

**Figure 2.**
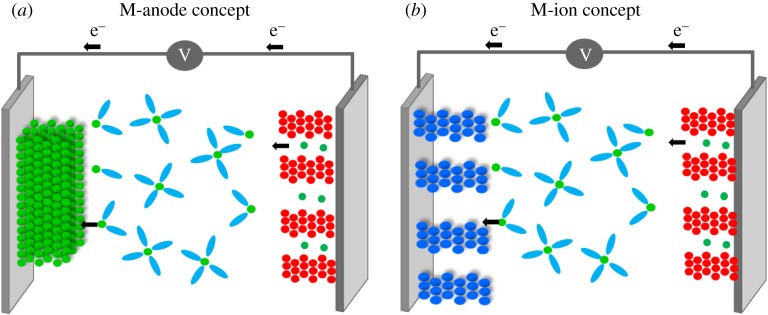
Schematic of a battery cell using a metal anode (*a*) or an insertion-type anode (*b*). (Online version in colour.)

**Figure 3.**
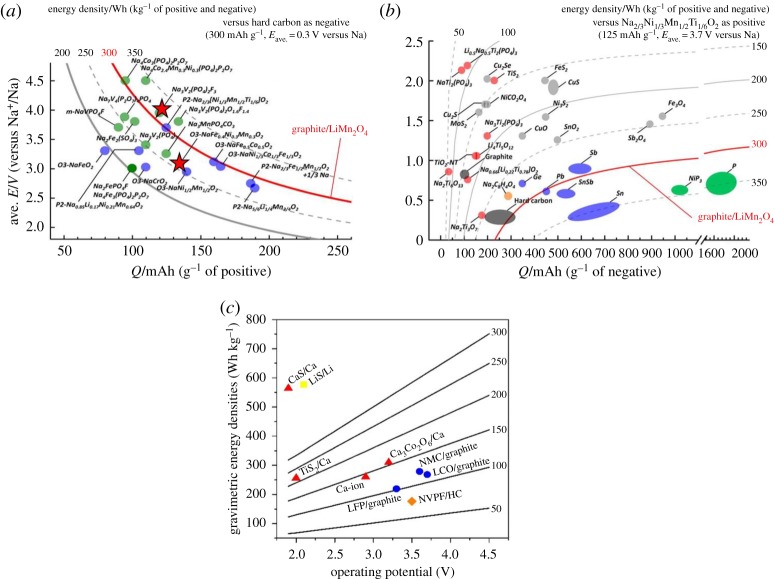
(*a*,*b*) Average voltage (*V*_ave_) and energy density (Wh kg^−1^) versus gravimetric capacity (mAh g^−1^) for selected positive electrode materials for NIBs. Reprinted from [11] with permission of the American Chemical Society. (*a*) Energy density was calculated with the hard carbon (reversible capacity of 300 mAh g^−1^ with *V*_ave_ = 0.3 V versus Na metal, as negative electrode materials. (*b*) Positive electrode is assumed to be Na_2/3_[Ni_1/3_Mn_1/2_Ti_1/6_]O_2_ for the energy density calculation. The stars correspond to prototypes reported in technical documents or press releases [13]. (*c*) Gravimetric energy densities for LIBs (circle), SIBs (diamond), Li–S (square) and estimated Ca (triangles) battery technologies. The straight lines are calculated energy densities of CaBs as a function of operation potential and capacities (denoted on the right of each line) [15]. (Online version in colour.)

In contrast with the M anode systems described above, M-ion concepts mimicking LIB technology but with alternative charge carrier ions instead of Li^+^ ions seem more straightforward, despite the use of intercalation host material anodes inducing a significant dead weight in the cell and penalizing energy density (figure 2). For such M-ion technologies, the main issue to consider is that the lowest negative electrode potential limit is set by the standard redox potential of the metal itself, which, when compared to lithium, is only somewhat lower for calcium, sodium and magnesium (approx. 170 mV, approx. 330 mV, approx. 670 mV, respectively), but significantly penalized for aluminium (approx. 1.38 V). Within this scenario, the most appealing M-ion case is the Na-ion technology, for which faster progress is expected as the *know-how* acquired for Li-ion can be easily imported in view of the chemical similarity of both concepts. Figures of merit comparable to those of Li-ion should be achievable at a lower cost and thus be suitable for larger scale applications [11–13]. However, results of simple estimates of the performance at cell level using open source models [14] indicate that Ca- or Mg-ion batteries would fall short in figures of merit with respect to Li-ion and would only be advantageous in terms of cost [15]. By contrast, divalent cation-based battery technologies using metal anodes would in principle allow for higher energy density (figure 3*c*). In this opinion piece, the state-of-the art, main challenges and research directions in the field of Na-ion, Mg- and Ca-based technologies will be discussed.

## 2. Na-ion

Progress in the sodium-ion battery (SIB) field is achieved at a quick pace, catalysed by the chemical analogies between lithium and sodium and the wide accumulated know-how of LIBs [11–13]. The materials choice on the negative side is relatively restricted, and today only hard carbon exhibits realistic application prospects [16]. The feasibility of SIBs using hard carbon negative electrodes is beyond any doubt, especially considering that the first generation of LIBs used hard carbon instead of graphite widely used today. However, the ability of this technology to compete in performance towards alternative contenders relies in overcoming practical bottlenecks. The most critical issue for hard carbon is the irreversible capacity loss upon the first cycle which severely penalizes the practical cell energy density achievable. This can at least partly be explained by the lower stability of the passivation layer formed at the negative electrode/electrolyte interface when compared with the one formed for LIB (commonly termed solid electrolyte interphase, SEI). Such passivation layer enables ionic transport and consists of electrolyte decomposition solid products. Since it avoids contact between electrolyte solvent molecules and the highly reducing negative electrode surface, it prevents further electrolyte reduction and its stability is essential for a proper functioning of the battery [17]. When compared with the case of LIB, the higher solubility of sodium salts results in significant interfacial instability issues for SIB. These result in accelerated electrolyte decomposition, which penalizes cycle life, and also possible migration of soluble species to the positive electrode promoting cell self-discharge [18,19].

With respect to the positive electrode, the chemical versatility of layered Na*_x_*MO_2_ oxides allows, by taking benefit from synergetic effects of both active and inactive metallic elements, to tune electrochemical performances to reach those of the positive electrode materials used in LIBs. Remaining challenges such as sensitivity of some of these compounds to the atmosphere, low operating voltages and limited reversible capacity could be solved by crystal chemical tuning of structure type and composition and even promoting the activation of anionic redox couple in addition to the conventional cationic ones [20]. Most of these drawbacks are solved using polyanionic-based compounds which, despite undoubtedly suffering from lower gravimetric capacities due to the presence of redox inactive elements (such as phosphate groups in Na_3_V_2_(PO_4_)_2_F_3_), clearly benefit from long-term cycling stability and fast ion diffusion thanks to stable and open three-dimensional frameworks [21]. Moreover, they typically exhibit higher operating voltages due to strongly covalent polyanionic groups [22,23]. The energy density for selected cell configurations is reported in figure 3*a*,*b*. For the first case, it is estimated considering hard carbon anodes and myriad possible cathodes, while for the second, it is calculated considering a layered NaMO_2_ (M = Ni, Mn, Ti) cathode and several possible anodes. Even if technical bottlenecks may exist to develop some of the configurations considered, the plots clearly evidence that NIB can be competitive with state-of-the art LIBs such as graphite/LiMn_2_O_4_ cells.

As for the choice of electrolyte, it could in principle be seen as a mere ‘blue-print’ from the LIB field. The dominant salt is NaPF_6_ and the solvents are typically mixtures of linear and cyclic alkyl carbonates (such as dimethylcarbonate (DMC) and ethylene carbonate (EC))—and even the optimal concentration is more or less the same as for LIBs: 1 M. The main difference with respect to the LIB field is the common use of propylene carbonate (PC) as solvent [24,25], which is not used in LIB as it co-intercalates in graphite used as negative electrode material causing its exfoliation. While other concepts have been suggested, including polymer electrolytes, the SIB future will most likely be based on organic liquid electrolytes. The use of co-solvents (for instance, ionic liquids) to enhance safety is also plausible [26]. Given a general increased attention to safety aspects, perhaps a non-fluorinated salt will be promoted, but this should in such a case most likely also happen simultaneously for LIBs. Indeed, to date the use of alternative salts has been found to result in corrosion of the aluminium foil used as current collector, which is compulsory for positive electrode materials operating at high potential.

Up-scaling from laboratory scale to larger cells is a necessary step to benchmark different battery chemistries against the state-of-the-art technologies, and most specifically SIBs versus LIBs, the main contender for most application prospects. Indeed, despite the environmental issues and the lower cost of sodium, the market prospects of SIBs will be related to both the overall cell cost, including active material synthesis and processing, and the practically attainable energy densities. Industrial R&D has been disclosed to some extent by European companies like Faradion (UK) or Tiamat (France) and some reported figures of merit marked in figure 3*a* seem to be comparable to state-of-the-art LIB.

## 3. Mg and Ca batteries

In contrast with the situation described above, multivalent ion-based batteries are in their early infancy [27]. Indeed, despite proof of concept achieved for Mg back in 2000 [28], commercialization is elusive due to a combination of technological bottlenecks. On the other hand, viability of a rechargeable technology based on calcium has only been considered recently, after reversible plating/stripping in organic electrolytes was achieved [29,30]. Most of the challenges in Ca- and Mg-based batteries arise from the high polarizing power of the divalent cations. Indeed, considering the ionic radius of Mg^2+^ (0.72 Å), Li^+^ (0.76 Å), Ca^2+^ (1.00 Å) and Na^+^ (1.02 Å), the charge density of divalent cations is considerably higher than that of monovalent ones. This leads to much stronger coulombic interaction between the divalent cations and their environment, both within the positive electrode active material and the electrolyte, resulting in sluggish migration and high desolvation energy barriers.

### (a) Metal anodes and electrolytes

In order to achieve energy densities higher than LIBs in Mg and Ca cells, the use of metal anodes is mandatory. Yet, rechargeability of metal anodes is very challenging, as exemplified the case of Li, which despite 40 years of research still shows significant issues related to safety, mostly linked to dendritic growth upon cell recharge [31]. For the case of Mg and Ca, the main issue is to find an electrolyte formulation allowing for high efficiency reversible metal plating and stripping. For any electrolyte candidate, a compromise has to be found between the use of solvents which are stable and do not form a passivation layer in contact with the naked metal (to the expense of less stability at high potential, such as THF), and solvents with higher stability at high potential for which stability in contact with the metal is kinetically achieved through the formation of a passivation film (SEI layer). This aspect is of particular interest, as the very slow diffusion of divalent ions through SEI layers [32,33] was suggested to be the main caveat in the development of secondary batteries operating at high potential. Indeed, some early success (by 1920) was achieved only when depositing magnesium from solutions in which no SEI was formed, as Grignard reagents (R-MgX, X = Br, Cl) in THF with only a very narrow useful potential window limited to less than 1.5 V versus Mg^2+^/Mg, low ionic conductivity and significant corrosion issues. Breakthroughs in the field were brought about by the intensive research carried out by the group of Prof. Aurbach during the last 20 years, which has been reviewed [8,9], and alternative electrolytes with wider electrochemical stability windows have been developed based on organometallic compounds in ethers (THF or glymes) in which no passivation layer is formed either. Such compounds are unfortunately highly corrosive, air-sensitive and nucleophilic, which precludes their use for practical batteries. Moreover, and in spite of recent advances in the field [34], the development of electrolytes enabling operation at potentials higher than 3 V remains an issue. Interestingly, some recent studies demonstrated the feasibility of Ca and Mg plating/stripping in the presence of a passivation layer built either upon cell operation or ‘artificially’ grown on the electrode prior to use [29,30,35]. Moreover, an electrochemical stability window over 4 V could be demonstrated for some cases [29,30], which opens optimistic prospects for the development of new electrolyte concepts. The nature of the solvation structure of Ca^2+^ and Mg^2+^ in the electrolyte is expected to play a major role in the overall kinetics of divalent cation-based batteries. Indeed, as a result of their highly polarizing character divalent, cations tend to exhibit stronger coulombic interactions with solvent molecules and exhibit higher coordination numbers in their primary solvation shell when compared with monovalent cations such as Li^+^ or Na^+^ [3]. Thus, desolvation energy is *a priori* expected to be higher for divalent systems, which induces an energetic penalty in the reactivity with the electrodes [3,36].

Interestingly, electrolytes in which salt concentration is high enough so that the amount of ions and solvent molecules is similar are now attracting considerable attention in the field, as these might lead to overcome some of the limitations mentioned above, despite some drawbacks related to high viscosity or wettability issues. These electrolytes exhibit a peculiar solvation structure (large amount of contact ion pairs and absence of free solvent molecules) and physico-chemical properties (electrochemical stability window broadening, change in the cation transport mode within the electrolyte) [37]. As an example, passivation of magnesium metal seems to be suppressed in highly concentrated Mg(TFSA)_2_/triglyme(G3)-based electrolytes (TFSA: bis (trifluoromethanesulfonyl) amide) (1 ≤ G3/Mg-salts ≤ 2)

Polymer electrolyte is an interesting candidate for multivalent cation-based systems since they consist of fixed anions along a polymeric backbone, thus resulting in much improved cation transport [38]. Son *et al*. [35] demonstrated a polymeric artificial interphase on Mg anode that extends the electrolyte stability range while enabling magnesium transport. Studies on few other polymer electrolytes were also reported, but Mg plating and stripping was not confirmed [39,40].

### (b) Cathodes

In analogy with LIBs and SIBs, the cathode materials for divalent battery concepts should (i) exhibit a redox centre, typically a transition metal, (ii) be able to reversibly react with the largest possible amount of charge carrier ions at high potential to have large energy density, and (iii) exhibit large cation migration rates to be able to operate under high power. The theoretical capacity of any material depends on the total number of electrons that can be transferred to the redox centre of the host and does not depend on the charge carrier ion. As a consequence, the number of divalent cations that have to react with the host to achieve a certain capacity is only half the number of lithium ions necessary for an equivalent charge transfer. Hence, should the compound be able to accommodate these more polarizing guest ions and reversibly at appreciable speed, much higher capacities would be achievable. The screening of positive electrode materials being strongly limited by the absence of standard electrolyte with adequate anodic stability, theoretical calculations using Density Functional Theory have been very useful in screening suitable electrode materials for divalent cation intercalation [9,41]. Yet, the topic is complex as some hosts that have been predicted to enable low migration barriers for calcium, such as a hypothetical CaMn_2_O_4_ spinel, come at the expense of phase stability and, hence, the polymorphs that can be synthesized experimentally are different and exhibit larger migration barriers [42].

The main challenge to overcome is sluggish solid-state diffusion for multivalent ions and the desolvation of solvent ligands at the cathode surface [9,41,43]. *A priori*, the use of more covalent electrode would be preferred, as the coulombic interactions with divalent ions would be less important. Indeed, proof of concept of Mg batteries was achieved using Chevrel phases (e.g. Mo_6_S_8_), which is still the best material available [9]. Compounds exhibiting open structural frameworks such as Prussian blue derivatives seem to also exhibit good power performances, despite complete elucidation of the role played by structural water in the redox mechanism is still elusive. In both cases, low operation potential induces a penalty in the achievable cell energy density. For the case of Ca, screening of materials has been more limited, as availability of electrolytes is much more recent, but trends do not seem to be significantly different than for Mg. There are only a few reports published in the early 2000s dealing with the feasibility of calcium intercalation, mostly limited to the study of V_2_O_5_. These indicate minor modifications of the structural framework, if any, upon reduction, which calls for caution in interpreting redox activity [44]. Indeed, translation of the know-how achieved by the academic community in the LIB field to multivalent systems is very tricky. The lack of in-depth knowledge about the electrolyte and anode behaviour, coupled to possible side reactions related to either corrosion, chemical compatibility issues or the presence of water as contaminant in the electrolyte (which may result in protons being the charge carrier ions and preclude the use of metal anode [45]), can lead to misleading assumptions with respect to the feasibility of divalent ion intercalation. Thus, the use of complementary characterization techniques is a must to clearly assess the abovementioned findings [46,47]. Co-intercalation of solvent molecules together with the charge carrier ion is sometimes observed for certain hosts (such as layered TiS_2_) [47] which can help in expanding van der Waals gaps and promote intercalation. This is most likely related to strong interactions between the cations and the solvent molecules which partially screen the charge and decrease coulombic interactions within the cathode solid framework. This has been suggested as a strategy to overcome limited migration within the solids, but the stability of the structure upon prolonged cycling involving such volume changes remains to be assessed [47,48].

Finally, it is worth to mention the hybrid battery concept which has been introduced recently to decouple issues related to metal plating and stripping from sluggish migration within the solid cathode framework due to strong coulombic interactions with divalent ions. In this cell design, a metal anode is used (for instance, Mg) and charge is compensated through the intercalation of a different cation (for instance, Li^+^ or Na^+^) at the cathode. Zhao and co-workers [49] proposed a Na–Mg hybrid battery using NaTi_2_(PO_4_)_3_ cathode and Mg metal anode.

Overall, systematic and thorough understanding of ion transport, solvation structure [3,50] and species present in the electrolyte and their role in the redox mechanisms are mandatory if multivalent ion-based batteries are to be turn from a laboratory curiosity into a commercial product.

## 4. Conclusion and perspectives

The fundamental understanding of the underlying structure–property relationships that govern battery performance achieved in recent times has prompted to a more rational battery technology development for which the ubiquitous Li-ion is the paradigm. This technology is by now quite mature and while still in progress at an incremental pace, it is widening its application scope from portable electronics to electric vehicles and also targeting large-scale grid applications. Nonetheless, efforts to develop alternative technologies have intensified in recent years due to peaks in the price of lithium coupled to geopolitical unstability of some producing countries. This seems to have attracted the interest of some large companies in Asia and start-ups in Europe and the USA and also interested the academic community, which sees an opportunity for rapid alternative developments stemming from the knowledge gained in Li-ion development.

While the current need to develop new battery technologies is beyond any doubt, the scientific community is pursuing a vast diversity of alternative strategies in the quest for high performance. Among these, Na-ion is one of the most extensively studied and the more similar to Li-ion. This should certainly bring about significant developments in the forthcoming years and maybe sharing of a significant part of large-scale storage developing applications for which it could become competitive with respect to Li-ion. Besides material research and optimization of each component separately, more comprehensive studies enabling the building of laboratory-scale prototypes have been carried out and a few companies are already developing the technology, which has very clear prospects for commercial viability.

In parallel, magnesium or calcium batteries hold promise for the applicability of metal anodes and hence large capacities. Nonetheless, the development of suitable cathodes is a must for the advent breakthrough in energy density, and, in turn, this requires novel electrolyte formulations enabling a wider electrochemical window. The power performance will likely not be comparable to any monovalent concept, due to the (inevitable?) sluggish guest ion diffusion in the cathode host materials. Such limitations, however, should not necessarily hamper implementation in application niches not requiring large power, especially if an efficient and cost-effective concept can be developed. Hybrid technologies in which a Mg or Ca metal anode could be coupled with a Li or Na insertion-type cathode could be an interesting configuration in order to improve metal anode-based battery power performances.

We foresee a quick and wide development and demonstration of the SIB technology together with an intensification of research in different multivalent chemistries in the near term (less than 5 years). These efforts should in a mid-term perspective (5–10 years) result in widespread commercialization of the former, most likely initially in large-scale grid-related applications, and determine the selection of viable systems and demonstration activities for the latter, which would then only be effectively developed and implemented in the much longer term (10–20 years).

Overall, materials science development in the battery field is still crucial and the current strategic efforts needed in the energy storage device optimization will certainly offer us opportunities to successfully unravel breakthroughs in the years to come.

## Data accessibility

This article has no additional data.
